# Acute Fulminant Cerebral Edema in a Child With Suspected Meningoencephalitis

**DOI:** 10.7759/cureus.45339

**Published:** 2023-09-16

**Authors:** Sara Monteiro, Beatriz Teixeira, Carolina Fraga, Andreia Dias, Ana Lúcia Cardoso, Daniel Meireles, Alzira Sarmento, Paula Regina Ferreira, João Silva, Cristina Garrido, Sara Gonçalves

**Affiliations:** 1 Paediatric Department, Centro Materno Infantil do Norte, Centro Hospitalar Universitário de Santo António, Porto, PRT; 2 Paediatric Department, Centro Hospitalar De Trás-Os-Montes E Alto Douro, Vila Real, PRT; 3 Paediatric Intensive Care Unit, Neonatology and Pediatrics Intensive Care Department, Centro Materno Infantil do Norte, Centro Hospitalar Universitário de Santo António, Porto, PRT; 4 Neurosurgery Department, Centro Hospitalar Universitário de Santo António, Porto, PRT; 5 Paediatric Neurology Unit, Centro Materno Infantil do Norte, Centro Hospitalar Universitário de Santo António, Porto, PRT

**Keywords:** hyperosmolar therapy, decompressive craniectomy, secondary intracranial hypertension, acute encephalitis, acute fulminant cerebral edema

## Abstract

Acute fulminant cerebral edema (AFCE) is a recently identified encephalitis type associated with significant morbimortality. Described as rare, limited data exists on its early detection and treatment. This paper describes a case of AFCE that progressed to unresponsive intracranial hypertension.

A previously healthy four-year-old boy presented with fever, myalgias, and neurological symptoms. Diagnostic assessments showed cerebrospinal fluid abnormalities, and despite medical interventions, his condition deteriorated rapidly and developed severe cerebral edema and herniation within 24 hours. A decompressive craniectomy was attempted to decrease intracranial pressure, without success. This case emphasizes the urgency of early AFCE recognition and effective management strategies given its severe prognosis, aiming to improve understanding and spur further research.

## Introduction

Acute fulminant cerebral edema (AFCE) is a recently recognized phenotype of encephalitis associated with significant morbimortality [1].

Acute encephalitis is more common in children and young adults, presenting with fever, headache, nausea, vomiting, photophobia, and stiff neck accompanied by signs of neurologic dysfunction. The prognosis is frequently favorable; when associated with AFCE it can rapidly become fatal or result in devastating neurological sequelae [2,3].

As AFCE appears to be uncommon, literature remains scarce regarding its early recognition and proper management [2]. The aim of this paper is to describe a case of AFCE that progressed to refractory intracranial hypertension, attempting to improve its characterization and stimulate further studies in this field.

## Case presentation

Presentation and initial management

**Figure 1:**
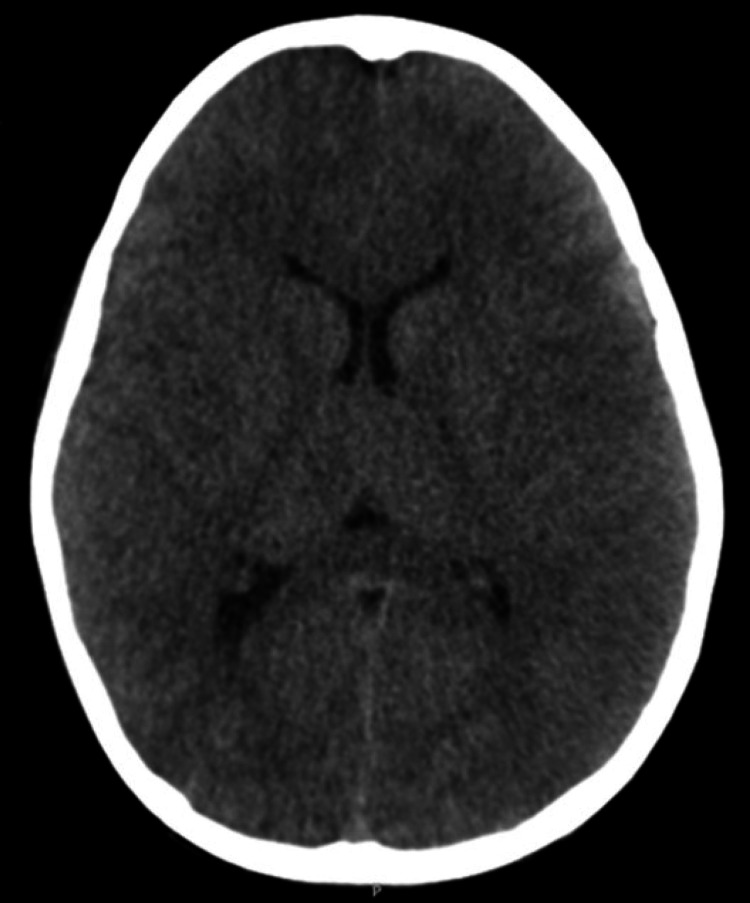
Head computed tomography scan revealing diffuse hypodensity of the brain parenchyma (diffuse dark areas), involving the cortico-subcortical parenchyma, the basal ganglia, and the thalamus.

A previously healthy four-year-old caucasian boy, with an updated national immunization schedule, was admitted to a secondary hospital pediatric emergency department (ED) with a one-day history of fever, difficulty feeding, progressive myalgias, and impaired walking. The child attended kindergarten and his sister had a recent upper respiratory tract infection, with no other epidemiologic context of illness. On admission, he presented a Glasgow coma scale (GCS) of 15, tender lower limbs, and dubious meningeal signs. Analytic workup revealed a serum glucose level of 70 mg/dl, leucocytosis with neutrophilia (20010/uL leukocytes, 16530/uL neutrophils, and 2180/uL lymphocytes) and a C-reactive protein of 51 mg/L. Chest x-ray was normal, the nasopharyngeal swab was negative for most frequent respiratory viruses [influenza A and B, respiratory syncytial virus, human bocavirus, rhinovirus, adenoviruses, enterovirus, parainfluenza virus 1-4, human metapneumovirus, coronavirus (229E, NL63, OC43), SARS-CoV-2], and Mycoplasma pneumoniae. The cerebrospinal fluid (CSF) analysis demonstrated pleocytosis (32 leukocytes/uL - 33% polymorphonuclear cells), glucose of 75 mg/dL, and proteins of 30 mg/dL. He was hospitalized and started empirical antibiotic therapy with ceftriaxone. On day three, he presented a worsening neurological status, gradually becoming drowsy and less responsive. Head computed tomography (CT) scan revealed loss of cortico-subcortical differentiation on the left parietal region, without signs of cerebral edema (Figure 1). Acyclovir and ciprofloxacin were added empirically. A few hours later, he initiated focal seizures (left conjugated gaze deviation and clonic right arm movements), treated with levetiracetam followed by sodium valproate.

**Figure 2:**
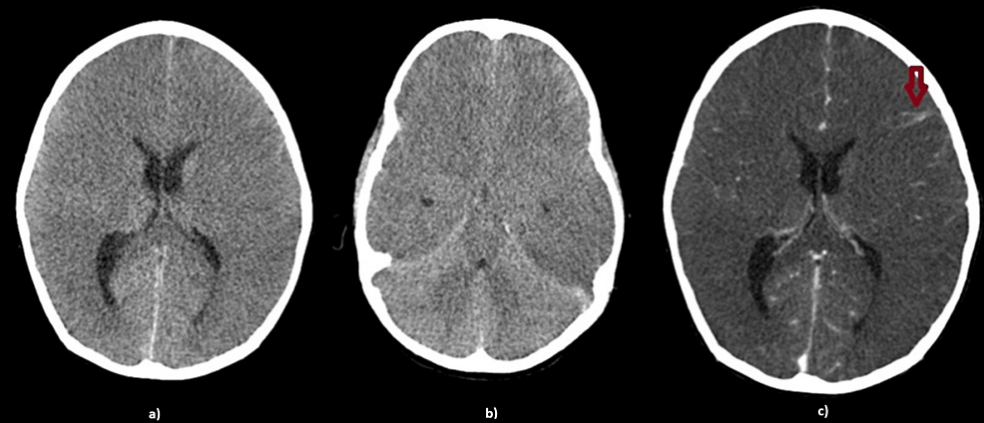
Head computed tomography scan showing a global effacement of the cerebrospinal fluid circulation spaces of the hemispheric sulci, convexity and basal cisterns, as well as a global loss of differentiation between gray and white matter in the supratentorial compartment (a and b). These findings are compatible with diffuse cerebral edema. After administration of contrast (red arrow on c), leptomeningeal contrast uptake is observed.

Due to a worsening state of consciousness (GCS 8), he was intubated, sedated with midazolam and fentanyl, and transferred to our pediatric intensive care unit (PICU). On admission, he was reactive to manipulation with isochoric, miotic, reactive pupils. EEG revealed global low amplitude and very frequent posterior left epileptiform activity, without criteria of epilepticus electrical status. Therapy with antibiotics, levetiracetam, midazolam, and fentanyl was maintained. After 24 hours, he suddenly presented with tachycardia (150-160 bpm), arterial hypertension (170/100 mmHg), and mydriatic, non-reactive pupils. Hyperosmolar therapy with 3% saline and hyperventilation were immediately started. An emergent contrast brain CT scan revealed diffuse cerebral edema, medial uncal herniation, and leptomeningeal contrast uptake (Figure 2). An intracranial pressure (ICP) sensor was placed with an opening pressure of 48 mmHg. Despite additional pharmacological measures to reduce ICP, namely dexamethasone, hyperosmolar therapy (3% saline continuous infusion and mannitol), and thiopental and analgesic optimization, ICP remained between 48-59 mmHg. Therefore, a bifrontal decompressive craniectomy was performed. Immediately after surgery, ICP normalised but mydriatic and non-reactive pupils persisted and he developed hyperthermia.

Post-operative management

**Figure 3:**
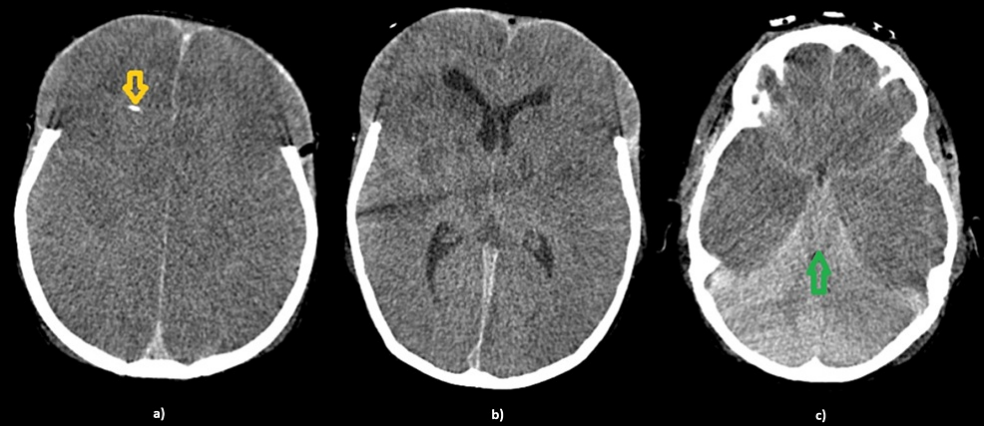
Head computed tomography imaging revealing pronounced global hypodensity in both cerebral hemispheres, absence of grey and white matter differentiation and complete effacement of the extra-ventricular supratentorial spaces. The brainstem mass effect is more exuberant, with a notable mesencephalic compression due to bilateral uncal herniation (green arrow on c). There is also a greater conflict of space in the posterior fossa. Brain parenchyma is herniated through the site of the bilateral frontal craniectomy. An intracranial pressure monitoring catheter (yellow arrow on a) is placed in the anterior horn of the right lateral ventricle.

After surgery, medical therapy included a 3% saline infusion, intravenous immunoglobulin (IVIG), and high-dose dexamethasone, maintaining sedation and analgesia. Approximately 48 hours post-surgery, ICP gradually increased to a persistent level of 32-33 mmHg despite mannitol, propofol, or thiopental administration, associated with refractory hyperthermia (core temperature 39.6-40ºC). A follow-up brain CT scan revealed significantly worsened diffuse cerebral edema (Figure 3). ICP remained elevated (maximum 60 mmHg) and cerebral perfusion pressure remained <50 mmHg despite inotropic therapy. Despite stopping levetiracetam and sedoanalgesia, he maintained a non-reactive coma and died in asystole in less than 48 hours.

Extensive infectious aetiological workup was negative, including viral and bacterial polymerase chain reaction (PCR) on CSF, culture of brain tissue collected intraoperatively, and respiratory bacterial and viral testing. Immunological studies including CNS antibodies and serum immunoglobulins were unremarkable. Histological and immunohistochemical examination of brain tissue revealed unspecific diffuse and severe cerebral edema.

## Discussion

We present a case of meningoencephalitis of unknown etiology that progressed to refractory AFCE.

Fulminant cerebral edema is defined as: (a) rapidly progressive elevated intracranial pressure and neurological deterioration (GCS < 8) and signs of brainstem dysfunction; and (b) abnormal results of impending cerebral herniation or herniation with brainstem compression in neuroimaging including computed tomography and magnetic resonance imaging [4-6].

Our patient fulfilled both criteria of the California Encephalitis Project (CEP) for encephalitis and AFCE (fever, altered mental status, and new-onset seizures, followed by progression to diffuse cerebral edema) [1].

This case is also consistent with AFCE reports, in which patients typically presented fever, seizures (most 24-48 hours before signs of AFCE [2]), and altered mental status [7,8]. However, the majority of children reported headaches and upper respiratory tract (URT) and/or gastrointestinal (GI) symptoms, absent in our patient.

Serum and CSF laboratory values of AFCE cases may be normal or slightly altered [1], as found in our patient, who presented with moderate pleocytosis. Despite ACFE can be triggered by common pediatric infections, no potential agents were identified in this case. Nevertheless, this is a recently described entity, without a clear etiology established. It is important to consider the possibility of a novel, unidentified agent that has not been ruled out in this case.

Our case aligns with the existing literature regarding the timing of cerebral edema onset, as most cases exhibit normal initial CT scans that later develop signs of edema within 24 to 48 hours [1,8].

The most critical reported complications include intracranial hypertension, hypoxic-ischemic encephalopathy, and herniation [1]. In this case, the first signs of ICP identified were sudden onset of arterial hypertension and fixed bilateral mydriasis.

There is limited data concerning invasive ICP monitoring indications and about the optimal medical and surgical approach in acute viral encephalitis [5]. A broad range of therapies has been described but no specific treatment was associated with an improved outcome of AFCE, such as intravenous immunoglobulin, intravenous high-dose methylprednisolone, or a combination of both (as used in our patient) [1].

Given the similarities to influenza-related neurologic complications, aggressive treatment of hyperthermia may be proven beneficial [1].

There is a lack of consensus about ICP management, particularly in AFCE. In most case series, hyperosmolar therapy and hyperventilation were used [1]. The efficacy of decompressive craniectomy remains uncertain, although sporadic use has been reported with variable outcomes, seeming to be especially effective when there is brain stem compression. Based on current evidence, this approach should be considered in cases refractory to medical management [2,6,9].

Regarding prognosis, the AFCE case series reports a fatal outcome in nearly 2/3 of patients, most within the first week of admission, the rest remaining with severe neurological sequelae, including persistent vegetative state [2,7].

## Conclusions

Acute fulminant cerebral edema in pediatric encephalitis is a rare, life-threatening condition. This report aims to contribute to literature enrichment, particularly for some particular presentation symptoms, such as the significant myalgias, rarely described in the literature, and for the unfavorable outcome, despite the performance of decompressive craniectomy.

Further studies are warranted to identify specific risk factors in patients with encephalitis who are prone to the development of AFCE, thus necessitating tailored attention and management. Additionally, there is a critical need for the development of more effective therapies.
